# Imaging Cellular Dynamics with Spectral Relaxation Imaging Microscopy: Distinct Spectral Dynamics in Golgi Membranes of Living Cells

**DOI:** 10.1038/srep37038

**Published:** 2016-11-22

**Authors:** Alireza Lajevardipour, James W. M. Chon, Amitabha Chattopadhyay, Andrew H. A. Clayton

**Affiliations:** 1 Centre for Micro-Photonics, Faculty of Science, Engineering and Technology, Swinburne University of Technology, Hawthorn, Victoria, Australia; 2 CSIR-Centre for Cellular and Molecular Biology, Uppal Road, Hyderabad, 500 007, India

## Abstract

Spectral relaxation from fluorescent probes is a useful technique for determining the dynamics of condensed phases. To this end, we have developed a method based on wide-field spectral fluorescence lifetime imaging microscopy to extract spectral relaxation correlation times of fluorescent probes in living cells. We show that measurement of the phase and modulation of fluorescence from two wavelengths permit the identification and determination of excited state lifetimes and spectral relaxation correlation times at a single modulation frequency. For NBD fluorescence in glycerol/water mixtures, the spectral relaxation correlation time determined by our approach exhibited good agreement with published dielectric relaxation measurements. We applied this method to determine the spectral relaxation dynamics in membranes of living cells. Measurements of the Golgi-specific C_6_-NBD-ceramide probe in living HeLa cells revealed sub-nanosecond spectral dynamics in the intracellular Golgi membrane and slower nanosecond spectral dynamics in the extracellular plasma membrane. We interpret the distinct spectral dynamics as a result of structural plasticity of the Golgi membrane relative to more rigid plasma membranes. To the best of our knowledge, these results constitute one of the first measurements of Golgi rotational dynamics.

Solvent or dipolar relaxation is a fundamental process that could be conveniently monitored in the condensed phase, where the rate of solvent (dipolar) reorientation is comparable to or slower than the fluorescence lifetime. Chemical as well as biological reactions can be influenced by the rate of dipolar relaxations. Examples range from charge transfer reactions to protein folding and membrane dynamics. From a cell biophysics perspective, one would like to have an experimental measure of these processes within the complex environment of the living cell with an overall goal of generating a dynamic map of the living cell.

Fluorescence offers a physical means to determine dipolar relaxation processes in the vicinity of a fluorescent probe. The emission spectrum of a fluorescent probe undergoes a detectable red shift from the initially excited Franck-Condon (or vertically excited) state to the solvated or relaxed state, if there is a dipole moment difference between ground and excited states. This spectral relaxation can be followed in real time using time-resolved fluorescence spectroscopy^1^,^2^. Alternatively, the presence of a red-shifted steady state emission spectrum upon excitation at the red edge of the absorption band (called red edge excitation shift or REES) is also a signature of dipolar relaxation on the fluorescence timescale^3^,^4^.

A unique feature of the cellular organization is morphological compartmentalization provided by cellular membranes. While the integrity of the cell as a whole is maintained by the outer plasma membrane, thereby providing the cell with its much needed individuality^5^, the intracellular matter remains spatially localized by a number of organelle membranes (such as Golgi, mitochondrial and nuclear membranes). Although the dynamics of the cellular plasma membrane has been studied by a number of approaches, relatively little information is available on the dynamics of intracellular organelle membranes. The Golgi apparatus is an organelle in the cell that plays an essential role in sorting, processing, modification and trafficking of proteins and lipids^6^. The Golgi apparatus consists of stacks of flattened disc-shaped membrane-bound compartments called cisternae, which are highly concentrated near the peri-centriolar region of the cell. The Golgi complex functions as a molecular factory in which proteins from the endoplasmic reticulum are chemically processed and sorted for transport to their eventual destination in the cell. Cholesterol concentration has been reported to increase progressively along the *cis*, *medial* and *trans* Golgi stacks, thereby providing a mechanism of protein sorting^7^. In addition, glycolipids and sphingomyelin are synthesized within the Golgi. The distinctive structure of the Golgi is not a static one but is instead maintained by a steady state dynamic equilibrium of membranes to and from other organelles in the cell. The remarkable structural plasticity and dynamic organization of this organelle eventually contribute to its complex function.

In this paper, we report the spectral relaxation dynamics in Golgi membranes and compare with dynamics in non-Golgi membranes in living cells. To achieve this, we use a Golgi-specific membrane probe, C_6_-NBD-ceramide^8^, which preferentially partitions into the membranes of the Golgi apparatus. We utilized fluorescence lifetime imaging microscopy with tuneable emission wavelength detection to determine the spatial distribution of the C_6_-NBD-ceramide probe and its associated excited state dynamics in single HeLa cells. Interestingly, we observed spectral relaxation dynamics in a sub-nanosecond timescale in Golgi membranes, distinct from the plasma membrane where the corresponding dynamics is in nanoseconds. We propose that these altered dynamics in Golgi membranes is related to the complex protein sorting function of the Golgi. To the best of our knowledge, these results constitute one of the early reports on rotational dynamics in Golgi using spectral relaxation imaging microscopy.

## Results

### Spectral relaxation in viscous solvents

**Figure 1:**
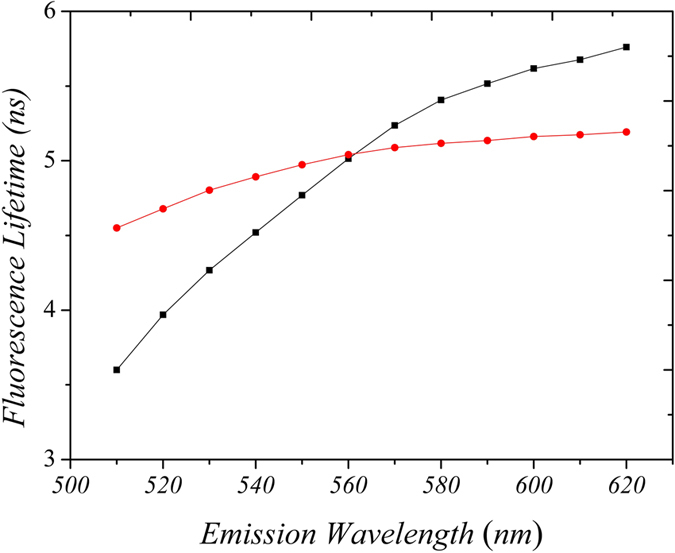
Fluorescence lifetime data for NBD-X in glycerol as a function of emission wavelength. Lifetimes were derived from the measured phase (τ_φ_, black) and the measured modulation (τ_m_, red) at a frequency of 35 MHz. Note the change in relative magnitude of τ_φ_ and τ_m_ at wavelengths greater than 560 nm. Temperature was 20 °C.

Molecules labelled with the NBD group has been extensively used as fluorescent probes to monitor membrane environment and dynamics due to a number of excellent photophysical properties of the NBD fluorophore^9^,^10^. To evaluate the behavior of the NBD fluorophore in a model solvent system, we measured the excited state decay of NBD-X dissolved in glycerol. Figure 1 depicts the phase lifetime (τ_φ_) and modulation lifetime (τ_m_) of NBD-X as a function of emission wavelength. Of particular note is the change in relative magnitude of the phase lifetime compared with the modulation lifetime as the detection wavelength is shifted from the blue to the red region (short to long wavelength) of the emission spectrum. For example, at wavelengths less than 560 nm, τ_φ_ is less than τ_m_, whereas τ_φ_ is greater than τ_m_ at wavelengths greater than 560 nm. As previously shown by Lakowicz and Balter^11^, this behaviour is characteristic of an excited state process, such as solvent relaxation.

**Figure 2:**
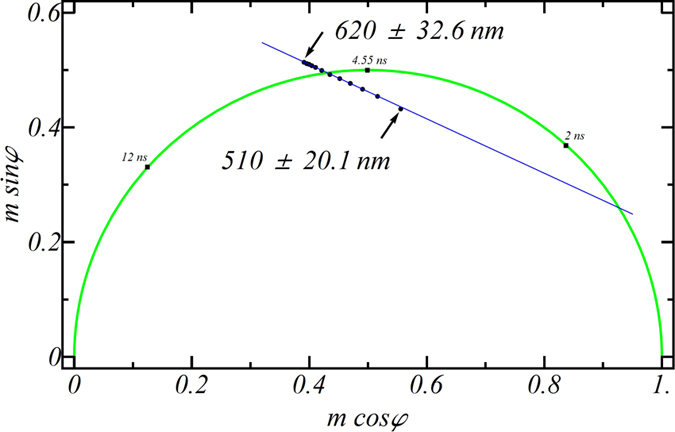
Phasor plot of NBD-X in glycerol for different detection wavelengths. Data corresponds to 11 FLIM measurements at detection wavelengths ranging from 510 to 620 nm with an increment of 10 nm. Solid line is a fit to a linear function (msinφ = −0.485*mcosφ + 0.704) with R^2^ = 0.999. Temperature was 20 °C.

**Figure 3:**
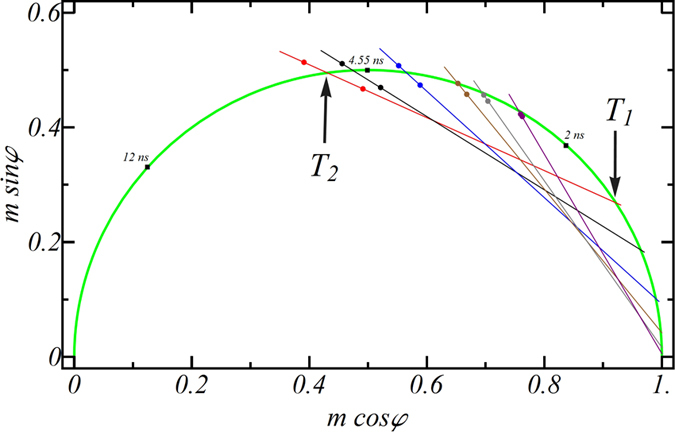
Phasor plot for NBD-X in solvents of varying viscosity. Data shown correspond to 100% glycerol (red line), 97% glycerol/3% water (black line), 90% glycerol/10% water (blue line), 80% glycerol/20% water (brown line), 70% glycerol /30% water (grey line), 50%/50% (purple line). In each set, there are two phasor points (one located outside and one inside of the semi-circle; shown in green) that correspond to detection wavelengths of 600 and 530 nm, respectively. Each of the fitted lines intersect the guiding semi-circle at two points (*e.g*., T_1_ and T_2_ for the red line, as shown in the graph). Temperature was 20 °C.

Figure 2 represents the data of Fig. 1 in the form of a phasor plot where x = mcosφ, and y = msinφ, and m is modulation and φ is phase. Data recorded from the red part of the emission spectrum exhibited an increased phase compared to the blue part of the spectrum, which is consistent with a spectral relaxation process (*i.e*., a phase delay in the emission from blue to red)^12^. Interestingly, data obtained from several wavelengths is well approximated by a straight line in phasor space which suggests a two state model is adequate to describe the excited state dynamics in this system. Due to this linearity (R^2^ = 0.999), we could use data from two wavelengths to extract the relevant decay dynamics (see Fig. 3).

To provide a quantitative estimate for the rates of spectral relaxation processes, we analyzed the data according to an approximate model for the relaxation processes. This model considers a time-dependent spectral shift in terms of detected emissions at 530 (I_530_) and 600 (I_600_) nm and an instrument correction factor G. The Generalized Polarization (GP) is given by:


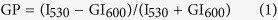


During spectral relaxation, GP will change from a value corresponding to the Franck-Condon spectrum (GP_o_) to that of the relaxed spectrum (GP_∞_). Assuming an exponential time course for the relaxation process with spectral relaxation correlation time T_s_, the time-dependent GP function is given by:


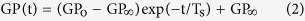


Assuming that the depopulation of the excited state is also exponential with lifetime T_2_ (*i.e*., I_total_(t) = (I_530_ + I_600_)(t) = I_o_ exp(−t/T_2_)), one can express the time-dependent emissions detected at 530 and 600 nm as:


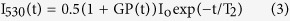



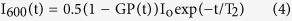


Substitution of equation (2) into equations (3) and (4) yields equations (5–7):


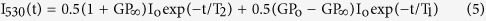



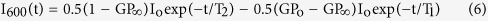



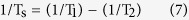


Equations (5) and (6) reveal that for the simplest model of spectral relaxation considered here, fluorescence dynamics consists of two relaxation times, T_1_ and T_2_, but with relative amplitudes which depend on the detection wavelength. In particular, this model predicts (for GP_o_ > GP_∞_) that data recorded in the blue side of the emission will exhibit double exponential decay in time with a phasor inside the universal circle. Data recorded on the red side of the emission will have a time-dependent rise and decay profile and a phasor outside the universal circle (see Fig. 2). In analogy with other systems displaying excited state dynamics^13^,^14^, T_1_ and T_2_ can be extracted from analysis of the phasor components recorded at 530 and 600 nm (equations 8, 9):


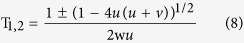



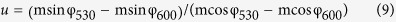



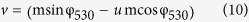


where w is the modulation frequency (w = 0.2512 ns^−1^ (40 MHz) or w = 0.22 ns^−1^ (35 MHz) for our experiments). Note that unlike the determination of the steady-state GP, which requires a G-factor, no G-factor is required to extract the time constants T_1_ and T_2_.

Carrying out this analysis for NBD-X in glycerol, the spectral relaxation correlation time (T_s_) was found to be 1.88 ± 0.04 ns. The value for T_s_ obtained from NBD-X excited state dynamics is in agreement (*i.e*., within 10%) with other reported values obtained using different approaches (T_s_ = 1.8 ns (NMR)^15^, T_s_ = 2 ns (dielectric relaxation)^16^, T_s_ = 1.98 ns (theoretical approach)^17^).

Figure 3 displays phasor plots for NBD-X in glycerol/water mixtures of differing composition recorded at 530 and 600 nm, and the corresponding relaxation times (T_2_ and T_1_) are shown. It is clear that adding water decreases the value of T_2_ and T_1_, and decreases the value of T_S_. This is in accordance with previous reports on polarity and hydrogen bonding decreasing the excited state NBD lifetime (decreasing T_2_)^18^ and decreased viscosity of glycerol (decreasing T_S_)^16^ with increasing water fraction. It is important to note that the phasors for NBD-X recorded at 600 nm for the higher water fractions (Fig. 3) lie at or below the universal circle, which does not strictly conform to the simple model proposed here. Thus the parameters obtained from these datasets should be treated as being approximate.

**Figure 4:**
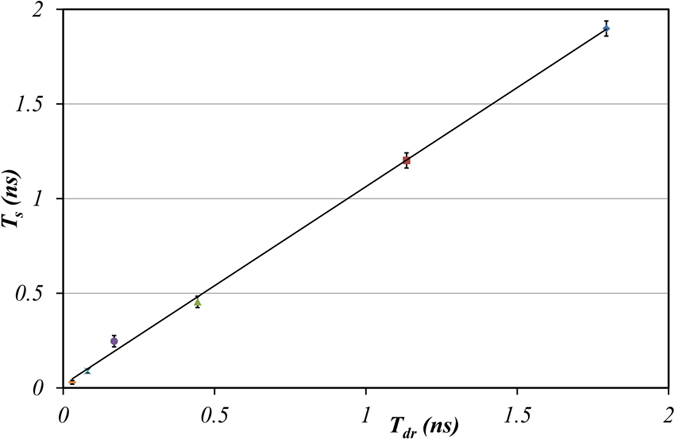
A comparison of solvent relaxation times derived using the phasor approach and from dielectric relaxation spectroscopy (literature). Data points represent experimental data and the solid line is a fit to a linear function. See text for more details.

A comparison of NBD-X spectral relaxation correlation time (T_s_) with literature data on glycerol/water mixtures using the principal dielectric relaxation time from dielectric relaxation measurements^16^ (T_dr_) is shown in Fig. 4. The regression line reveals good agreement between the two approaches (y = 1.05x + 0.02; R^2^ = 0.99 (Fig. 4)). Nevertheless because of the approximations in our method, we suggest that relaxation times greater than 0.5 ns can be reliably measured with our approach.

Taken together, these experiments in glycerol/water solution using a model NBD fluorophore demonstrate that our instrumentation and analysis can provide a quantitative measure of spectral relaxation in the time range of >0.5 ns with a NBD-linked biomolecule. We next turn to the application of these approaches to determining the spectral relaxation behavior of Golgi membranes and plasma membranes in living cells. For this purpose, we use C_6_-NBD-ceramide, which has been shown previously to be a specific probe for the membrane of Golgi^8^.

### Spectral relaxation of C_6_-NBD-ceramide in the Golgi and plasma membranes of living cells

**Figure 5:**
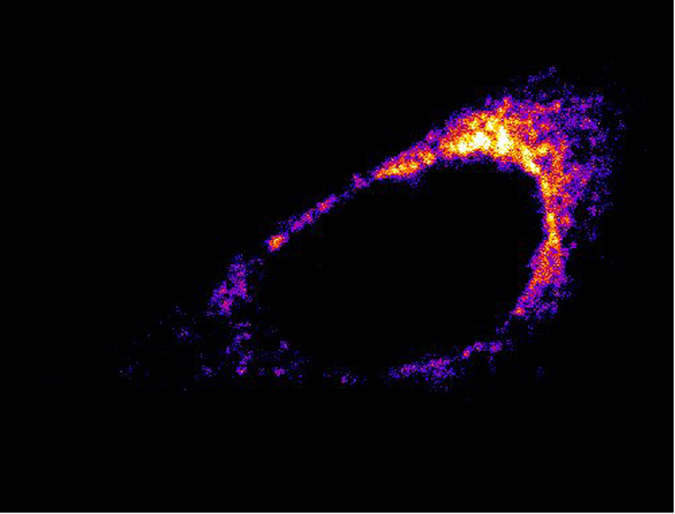
Cellular localization of C_6_-NBD-ceramide. Confocal image of Hela cell stained with C_6_-NBD-ceramide and then chemically fixed. The excitation wavelength was 488 nm.

**Figure 6:**
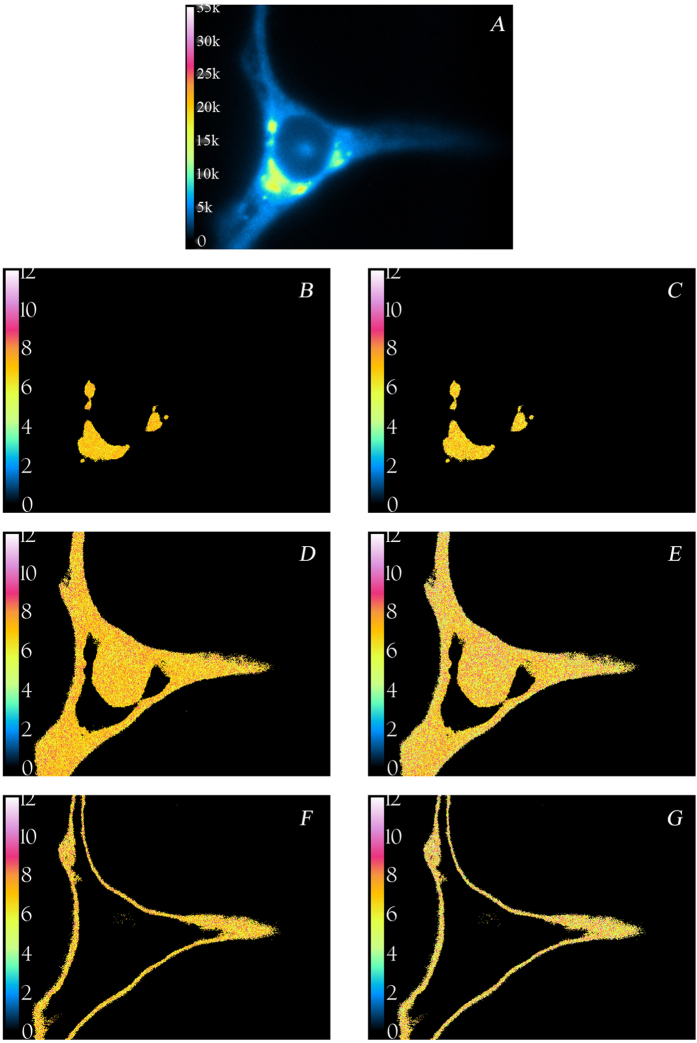
Fluorescence intensity and lifetime images of a HeLa cell labeled with C_6_-NBD-ceramide at the blue edge. Images were acquired with excitation at 470 nm and emission at 530 ± 20.2 nm. (**A**) Intensity image of cell (color table inset: low, 0 and high, 35,000 arbitrary units). Images in the left column represent modulation lifetime images and images on the right are phase lifetime images. Golgi membrane: (**B**) τ_m_ = 6.99 ns. (**C**) τ_φ_ = 6.79 ns. Golgi-excluded membranes: (**D**) τ_m_ = 6.94 ns. (**E**) τ_φ_ = 7.04 ns. Plasma membranes: (**F**) τ_m_ = 6.97 ns (**G**) τ_φ_ = 7.1 ns. The scale bar represents 10 μm. See text for other details. Temperature was 20 °C.

**Figure 7:**
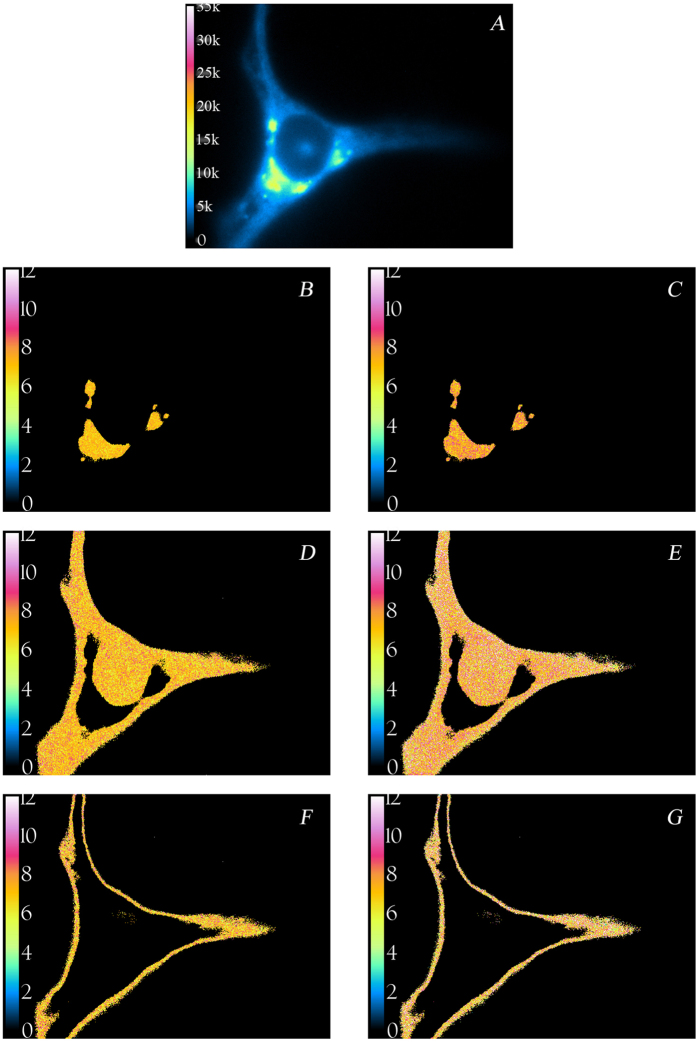
Fluorescence intensity and lifetime images of a HeLa cell labeled with C_6_-NBD-ceramide at the red edge. Images were acquired with excitation at 470 nm and emission at 600 ± 20.8 nm. (**A**) Intensity image of cell (color table inset: low, 0 and high, 35,000 arbitrary units). Images in the left column represent modulation lifetime images and images on the right are phase lifetime images. Golgi membrane: (**B**) τ_m_ = 7.08 ns. (**C**) τ_φ_ = 7.66 ns. Golgi-excluded membranes: (**D**) τ_m_ = 7.12 ns. (**E**) τ_φ_ = 8.12 ns. Plasma membranes: (**F**) τ_m_ = 7.2 ns (**G**) τ_φ_ = 8.13 ns. The scale bar represents 10 μm. See text for other details. Temperature was 20 °C.

Figure 5 shows a representative confocal laser scanning image of a HeLa cell stained with C_6_-NBD-ceramide. Fluorescence intensity and lifetime images of a typical HeLa cell stained with C_6_-NBD-ceramide obtained with our wide-field fluorescence lifetime imaging microscopy (FLIM) set-up are shown in Fig. 6. As can be seen in Fig. 6A, most of the probe is located in internal structures (resembling Golgi) and excluded from the nucleus. There is weaker fluorescence from the plasma membrane, which can be excluded by applying an intensity threshold. These observations are consistent with previous reports^19^. Figure 6B,C represents fluorescence lifetime images of the Golgi-stained membranes, Fig. 6D,E non-Golgi membranes, and Fig. 6F,G plasma membrane. When detected at 530 nm, the NBD-ceramide probe has a lifetime of about 7 ± 0.2 ns and the lifetime is largely independent of spatial location as determined from the phase or modulation of the emission. However, differences in fluorescence lifetimes are more evident for the data collected at 600 nm (Fig. 7). In the Golgi, non-Golgi and plasma membranes, the phase lifetime is significantly larger than the modulation lifetime (Δ0.6 ns, Golgi; Δ1.0 ns, non-Golgi; Δ1.0 ns, plasma membrane). This shows the spectral relaxation of C_6_-NBD-ceramide in HeLa cell membranes. In addition, the extent of relaxation appears to be different in Golgi membranes. Phasor plots corresponding to Figs 6 and 7 are shown in Figs S1–S4 (see Supporting Information).

**Figure 8:**
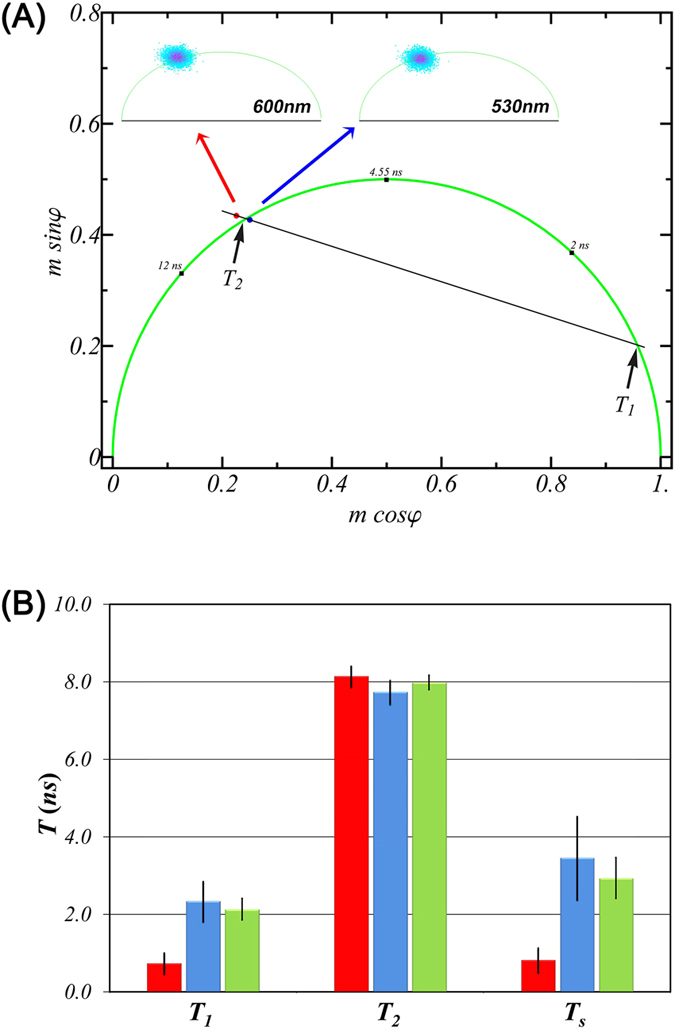
(**A**) Phasor (or polar or AB) plot representation of FLIM measurements. The inset diagrams and associated phasor clouds represent lifetime information from individual cells at pixel resolution. The averages of the phasor clouds (the region-averaged phasors) collected from the blue part (shorter wavelength) of the emission spectrum and the red part (longer wavelength) of the emission spectrum are depicted by the blue dot and red dot in the main diagram, respectively. A linear extrapolation is denoted by the black line with the two component lifetimes, T_1_ and T_2_ indicated by the positions of intersection of the line with the semi-circle. Note the position of one of the phasors is outside the semi-circle, indicative of excited state relaxation. (**B**) Derived lifetimes (T_1_, T_2_) and spectral relaxation correlation time (T_s_) from Golgi (shown as maroon bars), non-Golgi (blue bars) and plasma membrane (green bars) of 10 individual HeLa cells. The magnitudes of the columns are related to the average value while the error bars represent one standard deviation. Note the significantly faster spectral relaxation in the Golgi membrane (T_s_ = 0.8 ns) compared to the membranes in the non-Golgi (T_s_ = 3.5 ns) and plasma membranes of the cells (T_s_ = 2.9 ns). Temperature was 20 °C.

Figure 8 provides a representation of this analysis. In Fig. 8A, we have plotted the phasor clouds corresponding to the pixel-by-pixel data from cells recorded at 530 and 600 nm. The average values of these clouds are shown by the red and blue dots in the main plot in Fig. 8A. Values for T_1_, T_2_ and T_s_ from 10 individual cells are summarized in Fig. 8B. T_1_ was found to be significantly shorter in Golgi membranes (T_1_ = 0.8 ns) relative to the corresponding value in non-Golgi regions (T_1_ = 2 ns) or plasma membrane (T_1_ = 2 ns). However, T_2_ values were similar in all detected membranes (T_2_ ~ 8 ns). As a consequence, T_s_ in Golgi membranes is much smaller (T_s_ = 0.8 ns) than that in non-Golgi regions (T_s_ = 3.5 ns) or plasma membrane (T_s_ = 2.9 ns). This suggests that spectral relaxation in the membrane interface of Golgi is less restricted (faster) than non-Golgi membranes. These results could have significant implications in the context of Golgi dynamics and function.

## Discussion

Lateral dynamics in Golgi membranes has been previously studied^20^,^21^. On the other hand, rotational dynamics, a better indicator of protein-protein interaction and oligomerization due to weak dependence of lateral diffusion on the mass of the diffusing object in a membrane^22^, has not been previously explored in Golgi membranes. In this context, our results assume relevance and constitute a novel study in the overall area of organelle membrane dynamics.

We now turn to possible explanations for the observed spectral relaxation dynamics. One possible explanation for the observed spectral relaxation dynamics could be that the NBD probe is located in heterogeneous locations of the lipid bilayer, *i.e*., in a less polar, longer lifetime environment and a more polar, shorter lifetime environment. For example, NBD-ceramide has been reported to co-exist between liquid-ordered and liquid-disordered domains of membranes^23^. Herrmann’s laboratory^24^ has further shown that the excited state lifetime of NBD-C_6_ is about 11 ns in liquid-ordered domains and 7 ns in liquid-disordered domains. Because the polar environment in the liquid-disordered domains has a red-shifted emission relative to the non-polar environment in the liquid-ordered domains, this would give rise to an apparent spectral shift in time. However, because the blue emission in the liquid-ordered domains is longer lived (11 ns) than the red-shifted polar solvent emission from the liquid-disordered domains (7 ns), this would give rise to a longer phase in the blue part of the emission than in the red. The present data clearly show that the phase of the emission is larger in the red than the blue. A simple ‘population heterogeneity’ model therefore cannot account for our observations in membranes of living cells.

These observations are more consistent with a solvent relaxation model where the spectral relaxation time is related to relaxation of the solvent cage around the newly created NBD excited state due to the increase in dipole moment^25^ of the NBD group upon excitation. Trivial differences in level of hydration or polarity (as reflected in the lifetime values (T_2_) for Golgi and plasma membranes) cannot account for the differences in dynamics between Golgi and other membranes, since T_2_ values are essentially the same in both membranes. Therefore, the most likely conclusion is that the solvation dynamics in the different membranes are intrinsically different.

A possible contributing factor for faster solvent relaxation may lie in the metabolism of the C_6_-NBD-ceramide which is time-dependent and results in the production of NBD-cerebroside, NBD-sphingomyelin and the relocation of the probe from the Golgi to the plasma membrane^26^. Although the precise distribution of these different metabolites is unknown, having a different lipid tail may locate the probe in a different transverse or lateral location in the membrane where motional dynamics is altered. For example, a polarity gradient from non-polar to polar begins in the hydrocarbon region of the bilayer and extends out through the membrane interface to the water region. The lipid dynamics also exhibits a motional gradient but in the opposite direction with the membrane interface being most restricted and the hydrocarbon region least restricted^3^. Another possible contributing factor is the composition of the different membranes. The membrane composition of the Golgi^27^ and plasma membranes are in turn is related to their biogenic functions. The *cis*-Golgi is composed of thin bilayers with loose lipid packing and relatively low cholesterol content^7^. The loose lipid packing could contribute to faster overall rotational dynamics. The plasma membrane, in contrast, contains thick bilayers (due to high amount of cholesterol) with tight lipid packing related to its barrier function.

The analysis and results presented here indicate that spectral relaxation imaging microscopy promises to be a useful tool for quantitative imaging of spectral dynamics in complex environments. Our method provides quantitative estimates of excited state lifetimes and spectral relaxation correlation times from image data collected at two wavelength regions. The frequency-domain wide-field FLIM approach is characterized by fast acquisition, making it suitable for live cell imaging where reduction in light dose and tracking of processes in biological timescales are desired. It is important to emphasize that the analysis presented here is based on a simple model and only yields an apparent spectral relaxation time. For many systems undergoing spectral relaxation the correlation functions are typically non-exponential and characterized by the sum of two or more exponentially-decaying processes^28^,^29^. The interpretation of faster or slower dynamics could therefore also be interpreted as changes in the proportion of faster processes relative to slower processes. We have simulated the cases of two spectral relaxation times and one lifetime and found that the apparent spectral relaxation time obtained from our analysis is intermediate between the amplitude-weighted average of the two spectral relaxation times and the fluorescence-weighted average of the two spectral relaxation times (data not shown). We therefore believe that our model, although approximate, provides a useful parameter even in complex dynamic situations.

There is a wealth of studies on model membranes^30^,^31^,^32^,^33^,^34^,^35^,^36^ and an emerging literature^12^,^36^,^37^,^38^ on the use of dynamic imaging approaches to measure spectral relaxation in living cells. At the time this work was being completed, the Gratton laboratory^12^ published a phasor-based approach to measure membrane polarity and microviscosity. The phasor in the blue part of the spectrum was used to examine polarity or hydration, while the phasor in the green (longer wavelength) part of the spectrum was used to examine solvent dipolar dynamics^12^. Changes in polarity or microviscosity in living cells were inferred relative to measurements on model membranes of differing cholesterol content or phase state.

Further work is needed to understand dipolar solvent dynamics in membranes of living cells. A future goal is to understand the factors influencing solvent dynamics in complex environments and whether solvent relaxation can be used as a biosensor for detection of organelle-affected diseases. Since impairment of Golgi function appears to be linked to several diseases^39^, it is possible that these impairments could influence solvent relaxation times. At present, we are actively pursuing investigations along these lines.

## Methods

### Fluorescent membrane probe stock preparation

C_6_-NBD-ceramide (N-[6-[(7-nitro-2-1,3-benzoxadiazol-4-yl)amino]hexanoyl]-D-*erythro*-sphingosine) (Avanti Polar Lipids; Alabaster, AL) was used as a fluorescent lipid. 10 μl of chloroform solution of the fluorescent lipid was dried and dissolved in 1 ml DMEM to give a concentration of 5.75 μM of stock solution of C_6_-NBD-ceramide. The solution was vortexed and kept at 4 °C before use.

### Cell culture and treatments

HeLa Cells were cultured in a flask for 2 days in DMEM (+HEPES + 5–10% FCS + 1:100 Glutamate in 10% CO_2_) at 37 °C in CO_2_ incubator. After splitting, cells were freshly plated onto chambered coverglass (Lab-Tek II; Thermo Fisher Scientific; Rochester, NY) and were incubated for 1 day. Cells were first rinsed with phosphate buffered saline (PBS) twice. Cells were then labelled with C_6_-NBD-ceramide by incubating the cells for 30 minutes at 37 °C with a solution containing 1 ml DMEM and 40 μl of prepared stain solution (final concentration of 0.2 μM C_6_-NBD-ceramide). Prior to imaging, the cells were washed twice with DMEM.

### Solution experiments

The solvent relaxation of NBD-X (6-(N-(7-Nitrobenz-2-oxa-1,3-diazol-4-yl)amino)hexanoic acid) (AnaSpec Inc; Fremont, CA) was measured in glycerol and in glycerol/water mixtures. NBD-X was dissolved in glycerol at a concentration of 31.25 μM. Solutions of NBD-X in glycerol/water were prepared by mixing different volumes of water with the glycerol/NBD-X solution. NBD-X in glycerol/water mixtures contained 0, 3, 10, 20 30 and 50% (v/v) water.

### Fluorescence lifetime imaging microscopy

A Nikon microscope (Model Ti, Nikon, Japan) with Lambert instruments LIFA (Leutingwolde, The Netherlands) FLIM attachment was employed to measure FLIM images of HeLa cells. Samples were excited with sinusoidally-modulated (35 MHz) 474 nm light focused through a 100x/1.4NA oil objective and emission was observed through a hyper-spectral imaging system (His-400; Gooch & Housego; Orlando, FL) set at 530 ± 20.2 nm and 600 ± 20.8 nm, respectively. Twelve phase steps were recorded in pseudo-random order by using software provided by the manufacturer. Rhodamine 6 G in distilled water (lifetime = 4.1 ns) was used as a reference^40^. Lambert LI-FLIM software was used for analysis of experimental data. The phasor plots were exported using the Lambert LI-FLIM software. To measure lifetime of solutions in a cuvette, a 40x/0.7NA air objective was employed.

### Image analysis of FLIM data

The FLIM microscope produces wide-field images of fluorescence from the whole cell. Intensity thresholding was used to isolate fluorescence from the Golgi, non-Golgi or outer plasma membrane. C_6_-NBD-ceramide preferentially partitions to membranes of the Golgi apparatus but is also visible, to a lesser extent, in other cellular membranes including the plasma membrane. In this condition, non-Golgi membrane fluorescence was excluded with an intensity threshold to display pixels only with fluorescence signal above a certain threshold. Conversely, the plasma membrane regions were revealed by lowering the minimum intensity threshold and including an upper threshold on intensity values to exclude fluorescence from the internal membranes. Thresholding and FLIM analysis was performed using the LI-FLIM software.

### Confocal laser scanning microscopy

An Olympus FV1000 scanning laser confocal microscope was used to confirm Golgi localization of the C_6_-NBD-ceramide in HeLa cells. Excitation of the dye was provided with the 488 nm laser line focused through a 100x oil objective. Dye fluorescence was detected through a bandpass filter in the wavelength range 500–530 nm.

## Additional Information

**How to cite this article**: Lajevardipour, A. *et al*. Imaging Cellular Dynamics with Spectral Relaxation Imaging Microscopy: Distinct Spectral Dynamics in Golgi Membranes of Living Cells. *Sci. Rep*. **6**, 37038; doi: 10.1038/srep37038 (2016).

**Publisher’s note:** Springer Nature remains neutral with regard to jurisdictional claims in published maps and institutional affiliations.

## Supplementary Material

Supplementary Information
