# Supplementary material for: Imaging Cellular Dynamics with Spectral Relaxation Imaging Microscopy: Distinct Spectral Dynamics in Golgi Membranes of Living Cells
Source: Sci Rep. 2016 Nov 22;6:37038. doi: 10.1038/srep37038 (PMC5131650; doi:10.1038/srep37038)
Supplement: Supplementary Information [file srep37038-s1.doc]

**Supporting Information**

**Imaging Cellular Dynamics with Spectral Relaxation Imaging Microscopy: Distinct Spectral Dynamics in Golgi Membranes of Living Cells**

Alireza Lajevardipour,^†^ James W.M. Chon,^†^ Amitabha Chattopadhyay,^‡^,*

and Andrew H.A. Clayton ^†^,*

^†^Centre for Micro-Photonics, Faculty of Science, Engineering and Technology, Swinburne University of Technology, Hawthorn, Victoria, Australia

^‡^CSIR-Centre for Cellular and Molecular Biology, Uppal Road, Hyderabad 500 007, India

*Address correspondence to Amitabha Chattopadhyay, Tel: +91-40-2719-2578, Fax: +91-40-2716-0311, E-mail: [amit@ccmb.res.in](mailto:amit@ccmb.res.in); or Andrew Clayton, Phone: +61-3-9214-5719;

E-mail: aclayton@swin.edu.au


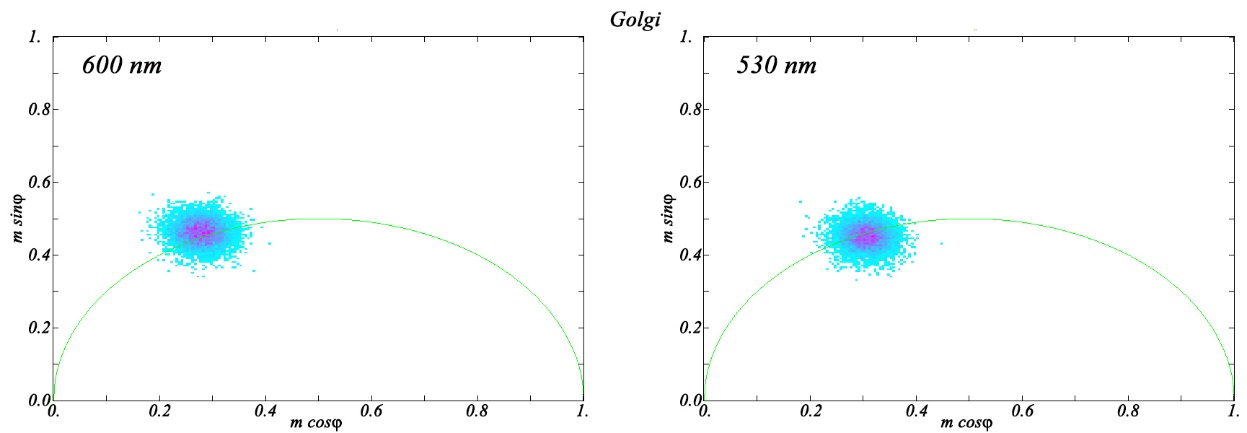


**Figure S1.** Phasor (AB or polar) plot of NBD-ceramide dynamic fluorescence in the Golgi membrane region of a single HeLa cell.


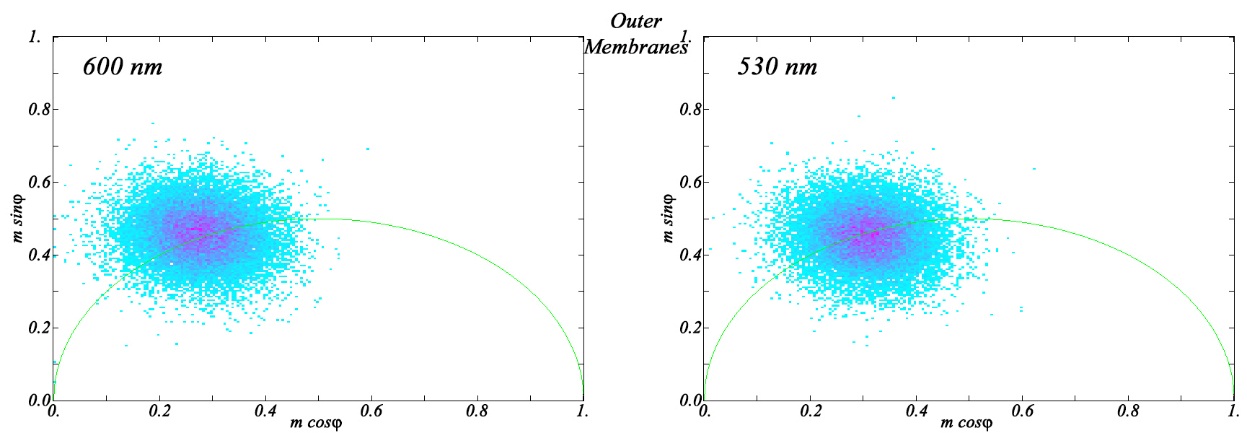


**Figure S2.** Phasor (AB or polar) plot of NBD-ceramide dynamic fluorescence in the plasma membrane region of a single HeLa cell.


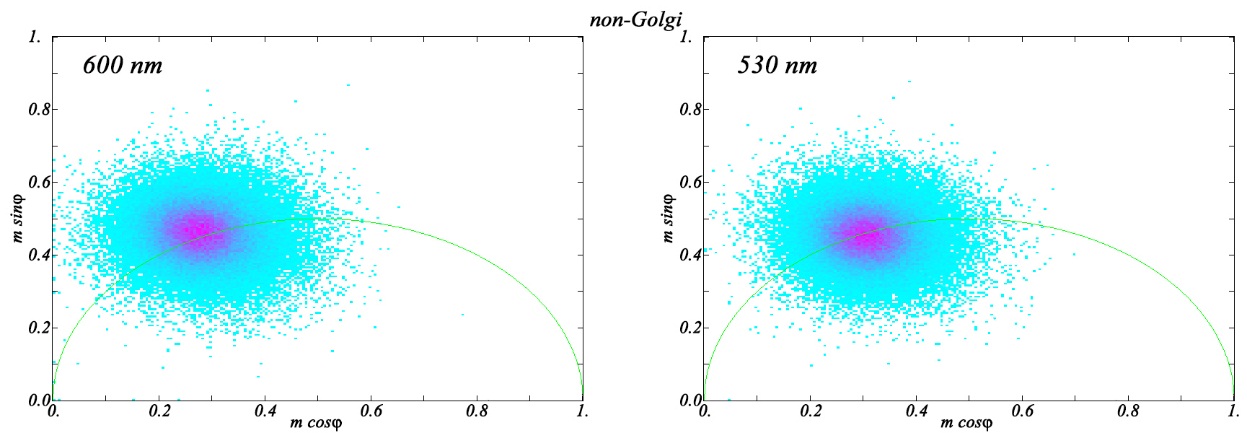


**Figure S3.** Phasor (AB or polar) plot of NBD-ceramide dynamic fluorescence in the non-Golgi membrane region of a single HeLa cell.


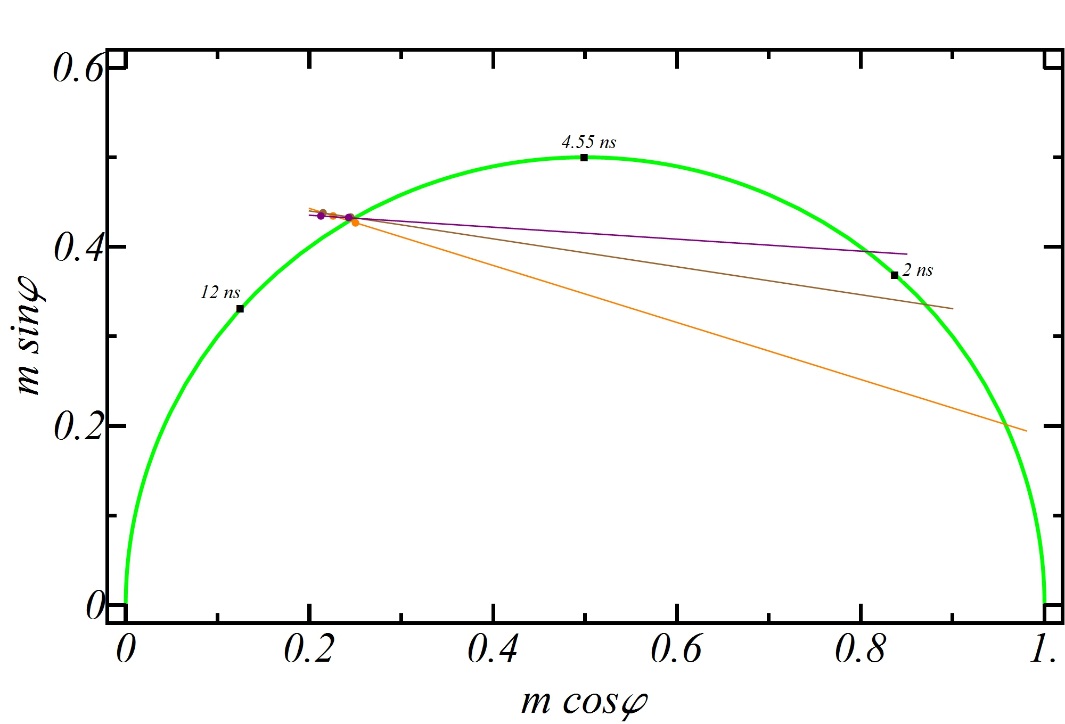


**Figure S4.** Representative Phasor (AB or polar) plot of NBD-ceramide dynamic fluorescence in the non-Golgi, Golgi and outer membrane region of a single HeLa cell. Each point represents an average (centroid) of the phasor cloud corresponding to a given region at a particular wavelength (see Figures S1-S3). Lines represent the extrapolation of data from red-edge and blue-edge of the emission. Note the distinct extrapolations for the three regions investigated showing region-specific membrane dynamics. Golgi (orange), Non-Golgi (brown) and plasma membrane (purple).
